# Transcriptomic Analysis Identifies Candidate Genes Related to Intramuscular Fat Deposition and Fatty Acid Composition in the Breast Muscle of Squabs (*Columba*)

**DOI:** 10.1534/g3.116.029793

**Published:** 2016-05-09

**Authors:** Manhong Ye, Bin Zhou, Shanshan Wei, MengMeng Ding, Xinghui Lu, Xuehao Shi, Jiatong Ding, Shengmei Yang, Wanhong Wei

**Affiliations:** * College of Bioscience and Biotechnology, Yangzhou University, Jiangsu Province, 225009, China; † College of Animal Science and Technology, Yangzhou University, Jiangsu Province, 225009, China; ‡ Jiangsu Co-innovation Center for Prevention and Control of Important Animal Infectious Diseases and Zoonoses, Yangzhou University, Jiangsu Province, 225009, China

**Keywords:** transcriptome, squab, intramuscular fat, fatty acid composition

## Abstract

Despite the fact that squab is consumed throughout the world because of its high nutritional value and appreciated sensory attributes, aspects related to its characterization, and in particular genetic issues, have rarely been studied. In this study, meat traits in terms of pH, water-holding capacity, intramuscular fat content, and fatty acid profile of the breast muscle of squabs from two meat pigeon breeds were determined. Breed-specific differences were detected in fat-related traits of intramuscular fat content and fatty acid composition. RNA-Sequencing was applied to compare the transcriptomes of muscle and liver tissues between squabs of two breeds to identify candidate genes associated with the differences in the capacity of fat deposition. A total of 27 differentially expressed genes assigned to pathways of lipid metabolism were identified, of which, six genes belonged to the peroxisome proliferator-activated receptor signaling pathway along with four other genes. Our results confirmed in part previous reports in livestock and provided also a number of genes which had not been related to fat deposition so far. These genes can serve as a basis for further investigations to screen markers closely associated with intramuscular fat content and fatty acid composition in squabs. The data from this study were deposited in the National Center for Biotechnology Information (NCBI)’s Sequence Read Archive under the accession numbers SRX1680021 and SRX1680022. This is the first transcriptome analysis of the muscle and liver tissue in *Columba* using next generation sequencing technology. Data provided here are of potential value to dissect functional genes influencing fat deposition in squabs.

Squab (young domestic pigeon under 4 wk old) has been consumed as a food by many nations for centuries. In China, squabs have been commercially raised on a large scale since the early 1970s (Tong *et al.* 2014). As the high nutritious value of squabs has become apparent to more and more people, the demand for them has rapidly expanded. In 2015, there were 30 million pairs of breeding pigeons in China (Cao and Chen 2015). With the development of the pigeon industry, squab production has progressively shifted from providing large amounts of protein to nourish populations, into promoting meats of consistent eating quality. The pigeon industry is beginning to consider breed differences in regard to nutritional values and meat quality, such as fatty acid (FA) composition, total lipids content, and tenderness, in addition to growth rate and meat yield (Bu *et al.* 2010; Zhang *et al.* 2012; Tong *et al.* 2014).

The total lipid content of muscle, intramuscular fat (IMF), and its FA composition play major roles in the quality attributes of meats, including sensory properties (flavor, juiciness, and tenderness) and nutritional values (Hocquette *et al.* 2010). It is generally assumed that IMF is important to induce a flavorful, juicy, and tender meat. The positive influences of IMF on the sensory quality traits of meat have been documented in pork (Gao and Zhao 2009), lamb (Watkins *et al.* 2013), and beef (Mateescu *et al.* 2015). As the unfavorable effects of dietary saturated FA exerted on health have been realized by an ever increasing number of consumers, strategies intending to modify the FA composition in meat have been applied in livestock to produce healthier products (Tous *et al.* 2013; Scollan *et al.* 2014). Up till now, breed-related differences in the capacity of lipid accumulation have been documented in pig (Wood *et al.* 2004a; Tyra *et al.* 2013), cattle (Gotoh *et al.* 2009), sheep (Qiao *et al.* 2008), and chicken (Wang *et al.* 2016). A number of single nucleotide polymorphisms (SNPs) significantly associated with the IMF trait of meat have been detected in functional genes involved in the lipid metabolism, which include the *FABP* (fatty acid-binding protein) (pig, Cho *et al.* 2011; beef cattle, Avilés *et al.* 2013), *LPL* (lipoprotein lipase) (chicken, Zhang *et al.* 2015), and *DAGT1* (diacylglycerol acyltransferase) (cattle, Wu *et al.* 2012). Meanwhile, effects of genetic variants on FA composition were also demonstrated (Taniguchi *et al.* 2004; Wood *et al.* 2008). SNPs that have an influence on the FA profile of meat have been identified in the *SCD* (stearoyl-CoA desaturase) (beef cattle, Li *et al.* 2012; goat, Avilés *et al.* 2016), *FASN* (fatty acid synthase) (cattle, Bartoň *et al.* 2016), and *LEPR* (leptin receptor) (pig, Li *et al.* 2010) genes. These intensively documented reports indicated the possibility of improving meat quality and its prohealth properties by increasing the IMF content and improving FA composition through genetic selection in livestock. However, comparatively little work has been conducted in squabs. Consequently, the biological mechanisms responsible for the deposition of fat in meat-producing animals (pig and cattle, especially) have been intensively explored, while limited information is available in squabs.

The development of the RNA-Sequencing (RNA-Seq) technique has opened new opportunities for researchers to obtain huge amounts of information about the transcriptome profile of tissues of interest, which help in the acquisition of knowledge concerning the mechanism of lipid deposition in livestock (Jeong *et al.* 2013; Chen *et al.* 2015; Wang Z. *et al.* 2015; Miao *et al.* 2015). To our best knowledge, there are no data available in the literature regarding the gene expression profiles in the muscle and liver tissues of squabs. In order to identify candidate genes that have effects on the IMF content and FA composition in the muscle of squabs, this present study was undertaken to: (1) demonstrate the differences in the genetic capacity of IMF deposition between squabs of different breeds; (2) analyze the FA profile of IMF in the breast muscle of squabs; and (3) identify candidate genes involved in the lipid metabolism that might be closely related to the IMF trait through comparison of transcriptome profiles of muscle and liver tissues between squabs with divergent IMF content. Results from our study will be helpful in understanding the background genetic mechanisms involved in IMF accumulation in squabs.

## Materials and Methods

### Ethics statement

All experiments with animals were performed following the “Guide for the Care and Use of Laboratory Animals” of the Comparative Medical Centre of Yangzhou University (a registered animal facility for supervising experiments on laboratory animals), and were in accordance with a protocol approved by the animal use committee of the Chinese Ministry of Agriculture.

### Animals and sample collection

All birds used in this study were reared under the same management system and processed in facilities operated by the same commercial supplier (Jiangnan Pigeon Industry Co. Ltd., Changzhou, China). Pigeons were kept in a stacked-cage raising system and squabs were housed within the same cage with both parents. A total of 68 female squabs, 34 from White Carneau (breed A) and 34 from Europigeon (breed B), were killed at 28 d of age by cervical dislocation. Live body weight of each bird was recorded. After euthanasia, roughly 1–2 g of pectoral muscle samples (on the left side) and liver samples were collected at necropsy and immediately flash frozen in liquid nitrogen. Breast muscles of both sides, including the pectoralis major and minor muscles, were removed from the carcasses (skinned and deboned), trimmed of visible adipose and connective tissues, placed into plastic bags individually, vacuum-sealed, and weighed. Left breast muscles were quickly frozen at −20° for later determination of intramuscular fat content and FA composition, which were undertaken not more than 1 wk later. Right breast muscles were submerged in ice for transport to the laboratory where they were subjected to 24 hr aging in a refrigerator (4°) prior to further analysis of pH, water-holding capacity, and tenderness.

### Determination of physical and chemical traits of breast muscles

Meats traits recorded in this study were determined in the Institute of Poultry Science, Chinese Academy of Agricultural Science, Yangzhou, Jiangsu, China.

The pH value of each breast muscle sample (pectoralis major) was measured 24 hr postmortem (pH_24_) at a depth of 1–2 cm using a portable pH meter (Testo 205, Testo AG, Lenzkirch, Germany) equipped with a penetrating electrode. Water-holding capacity (WHC) was calculated by difference in weight of a meat sample, ∼ 0.5 g, before and after being subjected to a pressure of 35 kg for 5 min. Warner−Bratzler shear force (WBSF) was measured on uncooked meat aged at 4° for 24 hr. Briefly, three rectangular samples (1 cm × 1 cm × 3 cm) were cut from each breast muscle parallel to the direction of muscle fibers. Tenderness was determined by WBSF measurement (C-LM3B, Tenovo, Beijing, China) by shearing meat samples perpendicular to the fiber direction. The shear force value was presented as the mean of the forces required to shear each set of samples and data were reported in Newtons. For each sample, pH_24_, WHC, and WBSF were determined from triplicate samples.

For the determination of IMF content, breast muscles were homogenized individually in a meat grinder after being thawed. About 1.5 g of each muscle homogenate was dried with sand to a constant weight at 103 ± 2° followed by cooling in a desiccator for at least 30 min. The IMF contents in breast muscles were measured by placing dried samples into a fat-free extraction sleeve and extracted in a Soxhlet device (VELP Scientifica, SER 148, Usmate, Italy) using anhydrous ether as the solvent, and results were expressed as percentages, on the basis of wet tissue weight.

The FA composition of IMF of the breast muscle was further investigated. FAs were transmethylated according to Morrison and Smith (1964). Analysis of fatty acid methyl esters (FAME) was performed with the gas chromatography (GC) method, using Agilent 7890A GC system equipped with a flame ionization detector and an Agilent J&W advanced capillary GC column (Agilent 122-2361, 60 m, 0.25 mm internal diameter, 0.15 μm film thickness). Samples were injected by an auto-sampler (Agilent Technologies 7683 Series, Santa Clara, CA). Fatty acids were identified by comparing the retention times with those of a standard FAME mixture, CDDE-GLC-NESTLE 37 FAME Mix (Nu-Check-Prep Inc., Elysian, MN). For each sample, GC analysis was carried out in duplicate and the results were expressed as percent of total FA methyl esters present in the sample. The sum of saturated fatty acid (SFAs), monounsaturated fatty acids (MUFAs), and polyunsaturated fatty acids (PUFAs) was also determined.

### Statistical analysis

All statistical procedures were performed by the SPSS 17.0 software package (SPSS Inc., Chicago, IL). An independent-samples *t*-test of the variance between two different pigeon breeds was carried out. Two-sided *P*-values of less than 0.05 were considered statistically significant. Results were given as mean ± SD in the text. The Pearson correlation coefficient between IMF content and WBSF value was estimated and significance was detected at the 5% level.

### RNA-Seq library construction for Illumina sequencing

The following protocols were performed by staff at the Oebiotech Co., Ltd., Shanghai, China. Three animals from each pigeon breed were used for the RNA-Seq analysis. Briefly, total RNA was isolated from the muscle and liver tissues using the TRIzol total RNA extraction kit (Invitrogen, Carlsbad, CA) according to the manufacturer’s instructions. The amount and purity of RNA were determined by NANODROP 2000c Spectrophotometer (Thermo Scientific) and the integrity was assessed with the Bioanalyzer 2100 (Agilent Technologies). Samples with RNA integrity number larger than 8.8 were considered acceptable for sequencing. mRNA libraries were constructed using the TruSeq RNA Sample Preparation Kit v2 (Illumina, Inc., San Diego, CA) according to the TrueSeq protocol and then sequenced in one lane using an Illumina Hiseq 2500 (Illumina Inc.) instrument.

### Data filtering and mapping of reads

The quality of the raw data was checked with FastQC v0.10.1 (http://www.bioinformatics.babraham.ac.uk/projects/fastqc/). Raw sequence reads with a Q-score < 20 and with a length shorter than 90 bp were removed for quality control and the remaining (clean) reads were mapped to the reference genome of pigeon (ftp://ftp.ncbi.nlm.nih.gov/genomes/all/GCF_000337935.1_Cliv_1.0) using bowtie 2 and TopHat v1.0.10 software (Trapnell *et al.* 2009). For functional annotation, the transcripts were compared against the reference genes (ftp://ftp.ncbi.nlm.nih.gov/genomes/all/GCF_000337935.1_Cliv_1.0/GCF_000337935.1_Cliv_1.0_rna.fna.gz).

### Differential gene expression analysis and functional annotation

The fragments per kilobase per million reads (FPKM) method was used to eliminate the influence of different gene length and sequencing level on the calculation of gene expression. The calculated gene expression was directly used for comparing the difference of gene expression between samples by the R package DESeq (http://bioconductor.org/packages/release/bioc/html/DESeq.html) (Anders and Huber 2010). If the FPKM value of either sample was zero, 0.01 was used instead of 0 to calculate the fold change. Genes with adjusted *P*-value of less than 0.05 and fold change > 1.5 were considered to be differentially expressed genes (DEGs). These DEGs were then subjected to Gene ontology (GO) annotation analysis (ftp://ftp.ncbi.nlm.nih.gov/genomes/all/GCF_000337935.1_Cliv_1.0/GCF_000337935.1_Cliv_1.0_genomic.gff.gz) and functional enrichment analysis using the classic algorithm and Fisher’s exact test. To further characterize the metabolic pathways of DEGs, the Kyoto Encyclopedia of Genes and Genomes (KEGG) database was used to analyze pathways (Kanehisa *et al.* 2008).

### Validation of RNA-Seq results

After total RNA was reverse-transcribed, quantitative real-time PCR (qRT-PCR) was performed on an ABI 7500 thermocycler (Applied Biosystems). The *GAPDH* (glyceraldehyde-3-phosphate dehydrogenase) was used as the internal reference gene for normalization of expression data (Wang Y. *et al.* 2015). Primers used in the present study are listed in Supplemental Material, Table S1. qRT-PCR in a 10-μl reaction volume with 0.1 μM of each primer (forward and reverse) and a quantity of cDNA corresponding to 10 ng of total RNA was performed using SYBR Premix Ex Taq reagents (Takara, Dalian, China). The validation performed on the same three samples per breed per tissue, which were used for RNA-Seq analysis, were run with three technical replicates. The PCR conditions were as follows: 2 min at 95° for the initial denaturation, followed by 40 cycles of denaturation at 95° for 5 sec, and 60° for 34 sec. The melt analysis curve was observed to confirm the amplification of single specific PCR fragments. Gene expression data were normalized to the *GAPDH*. Data from the relative quantification were transformed using the 2^−ΔΔCt^ method described by Livak and Schmittgen (2001). Significance was determined at *P* < 0.05.

The data from this study were deposited in NCBI’s Sequence Read Archive under the accession numbers SRX1680021 and SRX1680022. Supplemental materials included: Table S1, which contains the information on primer sequences used in qRT-PCR; File S1 and File S2, which contain the significantly enriched GO terms in the liver and muscle tissues, respectively; and File S3, which contains the detected significantly expressed genes involved in the lipid metabolism and the peroxisome proliferator-activated receptor (PPAR) pathway.

### Data availability

The data from this study were deposited in NCBI’s Sequence Read Archive under the accession numbers SRX1680021 and SRX1680022.

## Results

### Physical traits of the breast muscle

At the age of 28 d, no significant differences in live body weight (BW), breast muscle weight (BMW), and pH_24_ were observed between squabs from two breeds studied. However, significant differences were detected in other traits with respect to the ratio of breast muscle weight to live body weight (BMW/BW), WHC, and WBSF (shown in Table 1). Compared to birds from breed B, squabs from breed A had favorable meat traits in terms of a significantly higher proportion of breast muscle, better WHC, and lower value of WBSF (tender meat).

**Table 1:** Physical traits of the breast muscle recorded in the present study

	Breed A (*n* = 34)	Breed B (*n* = 34)	*P*-Value
BW (g)	484.15 ± 24.94	488.11 ± 24.62	0.512
BMW (g)	372.55 ± 52.66	350.59 ± 42.58	0.063
BMW/BW (%)*^a^*	76.86 ± 9.43	71.88 ± 8.37	0.024
pH_24_	6.05 ± 0.14	6.01 ± 0.18	0.202
WHC (%)*^a^*	79.59 ± 4.43	76.72 ± 6.25	0.033
WBSF*^a^* (Newtons)	11.54 ± 2.97	15.04 ± 2.87	0.000

Data are expressed as mean ± SD. Breed A, White Carneau; breed B, Europigeon. BW, live body weight; BMW, breast muscle weight; BMW/BW, the ratio of breast muscle weight to live body weight; pH_24_, pH value of the breast muscle 24 hr postmortem; WHC, water-holding capacity; WBSF, Warner−Bratzler shear force.

a Means differed significantly between squabs from two breeds (*P* < 0.05).

### Chemical traits of the breast muscle

A significant difference in the average IMF content was detected in the breast muscle samples from two pigeon breeds, 4.22 ± 0.77% and 3.61 ± 0.64% for breed A and breed B, respectively (*P* = 0.004). In this study, across all birds, there was a medium but significant (*P* < 0.001) correlation between WBSF value and IMF content (*r* = −0.517). As IMF content increased, less force was needed to cut through the meat.

Further analysis of the FA profile revealed that the main FAs identified in the breast muscle samples were palmitic (C16:0, 13.34–18.70%) and stearic (C18:0, 8.76–12.42%) acids for SFAs, palmitoleic (C16:1c9, 4.33–8.58%) and oleic (C18:1c9, 28.19–37.13%) acids for MUFAs, and linoleic (C18:2c9,c12, 13.65–18.64%) and eicosatrienoic (C20:3c11,c14,c17, 3.59–5.41%) acids for PUFAs. Differences between squabs from two pigeon breeds were significant when comparing concentrations (expressed as a percentage) of individual FAs in the IMF. In the breast muscle samples from breed A, the sum of SFAs was significantly lower, while the sum of MUFAs was significantly higher than that in breed B. The observed significant difference in the total content of MUFAs was mainly due to the significantly higher level of the predominant MUFA, oleic acid, in breed A. No significant differences were detected in the sum of omega 3 and omega 6 FAs or their ratio (shown in Table 2).

**Table 2:** Fatty acid composition of IMF from breast muscle samples of squabs

FAME	Breed A (*n* = 14)	Breed B (*n* = 14)	*P*-Value
C16:0	15.4895 ± 0.3850	15.3696 ± 0.2786	0.803
C18:0	10.0805 ± 0.2365	9.7712 ± 0.2076	0.335
C10:0	0.1157 ± 0.0178	0.1507 ± 0.0125	0.119
C12:0	0.1527 ± 0.0263	0.1727 ± 0.0244	0.583
C13:0	0.1092 ± 0.0138	0.1239 ± 0.0048	0.323
C14:0*^a^*	0.2913 ± 0.0315	0.3779 ± 0.0251	0.041
C15:0*^a^*	0.1157 ± 0.0141	0.2350 ± 0.0504	0.038
C17:0*^a^*	0.7085 ± 0.0694	1.0627 ± 0.0992	0.007
C20:0*^a^*	0.6875 ± 0.1316	1.1255 ± 0.0710	0.008
C21:0	1.0505 ± 0.0967	1.1094 ± 0.0335	0.570
C22:0	0.6779 ± 0.0373	0.7694 ± 0.0445	0.127
C23:0	0.4894 ± 0.0442	0.6581 ± 0.0985	0.130
C18:1c9*^a^*	32.2607 ± 0.6585	30.6222 ± 0.4520	0.050
C16:1c9	6.4010 ± 0.2891	6.6256 ± 0.1040	0.471
C14:1c9*^a^*	0.0979 ± 0.0099	0.1926 ± 0.0236	0.001
C15:1c10	0.9772 ± 0.0484	0.8852 ± 0.0683	0.282
C17:1c10	1.1355 ± 0.2063	1.0084 ± 0.1314	0.608
C18:1t9	0.7260 ± 0.1073	0.5854 ± 0.1330	0.418
C20:1c11	1.3144 ± 0.1551	1.2893 ± 0.1505	0.908
C24:1c15	0.8442 ± 0.0350	0.9206 ± 0.0308	0.113
C18:2c9,c12	15.4767 ± 0.4062	15.5508 ± 0.3440	0.890
C18:2t9,t12	0.4280 ± 0.2789	0.1940 ± 0.0459	0.415
C18:3c6,c9,c12	1.6038 ± 0.2583	1.3369 ± 0.0764	0.331
C20:2c11,c14	0.4693 ± 0.0518	0.5959 ± 0.0375	0.058
C20:4c5,c8,c11,c14*^a^*	0.3997 ± 0.0492	0.7141 ± 0.0731	0.001
C22:2c13,c16	0.6902 ± 0.1129	0.8465 ± 0.0677	0.246
C18:3c9,c12,c15	1.1975 ± 0.0850	1.3377 ± 0.0537	0.175
C20:3c11,c14,c17	4.5368 ± 0.1443	4.6063 ± 0.0715	0.671
C20:5c5,c8,c11,c14,c17	0.4261 ± 0.0271	0.4433 ± 0.0239	0.639
C22:6c4,c7,c10,c13,c16,c19	0.3612 ± 0.0336	0.4525 ± 0.0328	0.063
∑SFA*^a^*	29.9684 ± 0.2561	30.9258 ± 0.3636	0.041
∑MUFA*^a^*	43.7568 ± 0.5990	42.1293 ± 0.4501	0.039
∑PUFA	25.5894 ± 0.5442	26.0779 ± 0.3057	0.441
P/S	0.8548 ± 0.0203	0.8450 ± 0.0148	0.698
∑n-6	19.0677 ± 1.6118	19.2381 ± 0.9752	0.738
∑n-3	6.5217 ± 0.8445	6.8398 ± 0.4088	0.216
n-6/n-3	2.9695 ± 0.4736	2.8200 ± 0.1902	0.283

Values are presented as mean ± SD. Breed A, White Carneau; breed B, Europigeon. ∑SFA, total saturated fatty acids; ∑MUFA, total monounsaturated fatty acids; ∑PUFA, total polyunsaturated fatty acids; P/S, the ratio of PUFAs to SFAs; ∑n-6, total omega 6 fatty acids; ∑n-3, total omega 3 fatty acids.

a Means differed significantly between two breeds (*P* < 0.05).

### Transcriptome analysis and DEGs screening

**Figure 1:**
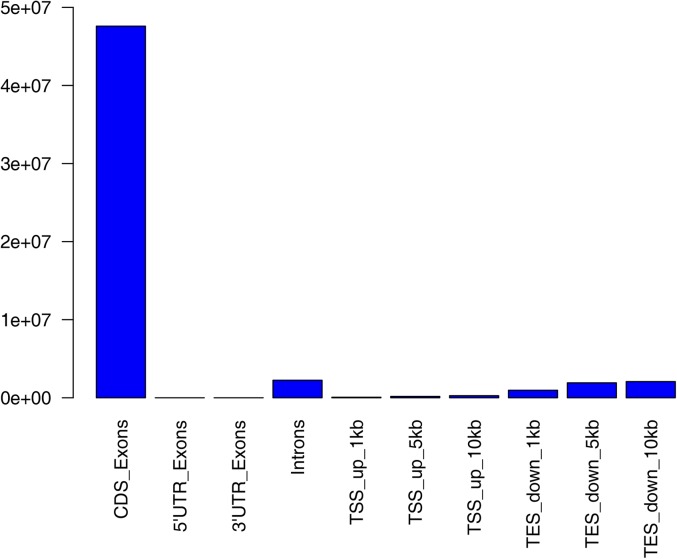
Detailed distribution of reads mapped to the reference genome. CDS, coding sequence; TES_down, downstream of the transcription ending site; TSS_up, upstream of the transcription starting site; UTR, untranslated regions.

To identify DEGs related to the differences in IMF content in the breast muscle, RNA-Seq was used to obtain the transcriptomes of liver and muscle tissues from squabs of two pigeon breeds. Detailed results of sequencing and assembly are shown in Table 3. A total amount of 603.58 million reads with an average length of 125 nucleotides was acquired from the RNA-Seq experiment. On average, 51.71 ± 6.59 and 48.88 ± 3.20 million reads were obtained for libraries from liver and muscle samples, respectively. About 73.06% (70.50–75.93%) of reads were mapped to the reference genome, of which 96.91% mapped to unique genomic locations. An average of 60.44% of the mapped reads corresponded to annotated genes, 90.03% (88.55–91.26%) of them were located in exons, and 4.43% (4.10–4.95%) were in introns. The detailed distribution of reads is shown in Figure 1.

**Table 3:** Summary of Illumina sequencing

Tissue	Sample Name	Raw Reads	Clean Reads	Clean Bases (bp)	Valid Ratio (%)	Q30 (%)	GC Content (%)	Total Mapped Reads
Liver	A14G	54272912	53984128	6743324339	99.39	92.50	49.0	37007492
Liver	A17G	54897634	54612062	6821811425	99.41	92.62	49.0	37514969
Liver	A24G	58981376	58670858	7328755933	99.40	92.40	49.0	41828564
Liver	B6G	43045126	42823256	5349250748	99.41	92.62	49.0	29871220
Liver	B14G	56363624	56062464	7003011885	99.39	92.55	48.5	38557847
Liver	B15G	44367286	44136162	5513068527	99.40	92.27	48.0	30338161
Muscle	A14M	51679634	51416892	6421765893	99.40	91.69	49.0	25474058
Muscle	A17M	46154200	45912278	5735008428	99.40	92.32	49.0	23944779
Muscle	A24M	54084880	53808252	6721243425	99.41	92.30	50.0	28855995
Muscle	B6M	46911708	46665656	5829074439	99.40	92.34	49.0	23568399
Muscle	B14M	49465288	49209082	6146719244	99.41	92.28	49.0	25481567
Muscle	B15M	46522126	46280970	5781030700	99.41	92.29	49.0	24009210

A, White Carneau; B, Europigeon.

The mean gene expression in the muscle and liver tissues between squabs from two breeds was highly correlated (*P* < 0.0001). The Pearson correlation coefficient was 0.99 and 0.97 for the muscle and liver tissue, respectively, showing that most of the genes had a similar behavior. Using the DESeq program, 1027 transcripts were identified as significantly differentially expressed in the liver tissue between two breeds, of which, 372 transcripts (mapped to 224 unique genes) were up-regulated and 655 transcripts (mapped to 412 unique genes) were down-regulated in breed A. In the muscle tissue, a total of 767 DEGs were identified, of which, 268 transcripts (mapped to 151 unique genes) were up-regulated and the remaining 499 transcripts (mapped to 298 unique genes) were down-regulated in breed A. These unigenes were successfully categorized into the three main GO categories of biological process, cellular component, and molecular function. The proportion of these DEGs categorized into GO biological process of lipid metabolic and regulatory process was 9.4% (60/636) and 5.6% (25/449) in liver and muscle tissues, respectively. They were involved in GO terms such as medium- and very long-chain fatty-acyl-CoA metabolic process, triglyceride homeostasis, FA oxidation, and phospholipid biosynthetic process. Significantly enriched GO terms in DEGs were shown in File S2 (liver) and File S3 (muscle).

In the present study, the KEGG database was used to analyze the DEGs in the metabolic pathway. Since the IMF content of muscle results from the balance between uptake, synthesis, and degradation of lipids, we focused mainly on the pathways directly involved in the lipid metabolism (Table 4). After the zero-expression genes were removed from the analysis, a total of 27 unique DEGs involved in 12 different lipid metabolic pathways were identified in liver. The fold changes for those DEGs ranged from 1.47 to 11.27. We noticed that two DEGs also belonged to the PPAR signaling pathway, which was considered to be one of the most important pathways involved in the regulation of lipid metabolism. In the muscle tissue, 16 DEGs involved in 12 different lipid metabolic pathways were identified. The fold changes for those DEGs ranged from 1.62 to 8.12. Four of these DEGs were also involved in the PPAR pathway. Including these six DEGs, a total of 10 significant DEGs were identified from the PPAR pathway with fold changes ranging from 1.64 to 11.27. Most of them (7/10) were significantly up-regulated in liver and/or muscle tissues from breed A. Significant DEGs involved in the lipid metabolism were listed in File S3.

**Table 4:** Significant DEGs involved in the pathways of lipid metabolism

Pathway		NCBI Reference Sequence (Expressed in Liver)	NCBI Reference Sequence (Expressed in Muscle)
path:clv00072:	Synthesis and degradation of ketone bodies	XM_005511687.1,XM_005511691.1, XM_005514550.1	
path:clv00120:	Primary bile acid biosynthesis	XM_005500333.1,XM_005501950.1, XM_005501951.1, XM_005506223.1,XM_005506490.1	XM_005506223.1
path:clv00561:	Glycerolipid metabolism	XM_005498222.1,XM_005500903.1, XM_005501648.1, XM_005506697.1,XM_005509797.1,XM_005510541.1	XM_005499093.1, XM_005499094.1, XM_005500903.1,
path:clv00061:	FA biosynthesis	XM_005511837.1	XM_005505177.1, XM_005513915.1, XM_005515450.1
path:clv00071:	FA degradation	XM_005498018.1, XM_005498222.1, XM_005506697.1, XM_005511837.1	XM_005505177.1, XM_005513915.1, XM_005515450.1
path:clv01040:	Biosynthesis of unsaturated FAs		XM_005511038.1
path:clv00100:	Steroid biosynthesis	XM_005509160.1	XM_005513640.1
path:clv00564:	Glycerophospholipid metabolism	XM_005500903.1, XM_005501648.1, XM_005503907.1, XM_005510333.1, XM_005510541.1, XM_005512011.1	XM_005500903.1, XM_005512626.1
path:clv00140:	Steroid hormone biosynthesis	XM_005498168.1, XM_005500333.1, XM_005504843.1, XM_005506490.1,XM_005509223.1	XM_005504843.1
path:clv00600:	Sphingolipid metabolism	XM_005500903.1, XM_005505197.1, XM_005508143.1, XM_005509734.1	XM_005500903.1, XM_005505196.1, XM_005505197.1, XM_005508140.1, XM_005508142.1, XM_005508143.1, XM_005510696.1
path:clv00590:	Arachidonic acid metabolism	XM_005503412.1	XM_005498904.1, XM_005513677.1
path:clv00565:	Ether lipid metabolism	XM_005500903.1, XM_005507270.1, XM_005512011.1	XM_005500903.1, XM_005507271.1
path:clv03320:	PPAR signaling pathway	XM_005498269.1, XM_005498270.1, XM_005502057.1, XM_005506490.1, XM_005511837.1, XM_005513300.1	XM_005499093.1, XM_005499094.1, XM_005500050.1, XM_005502057.1, XM_005505177.1, XM_005513915.1, XM_005515450.1

**Figure 2:**
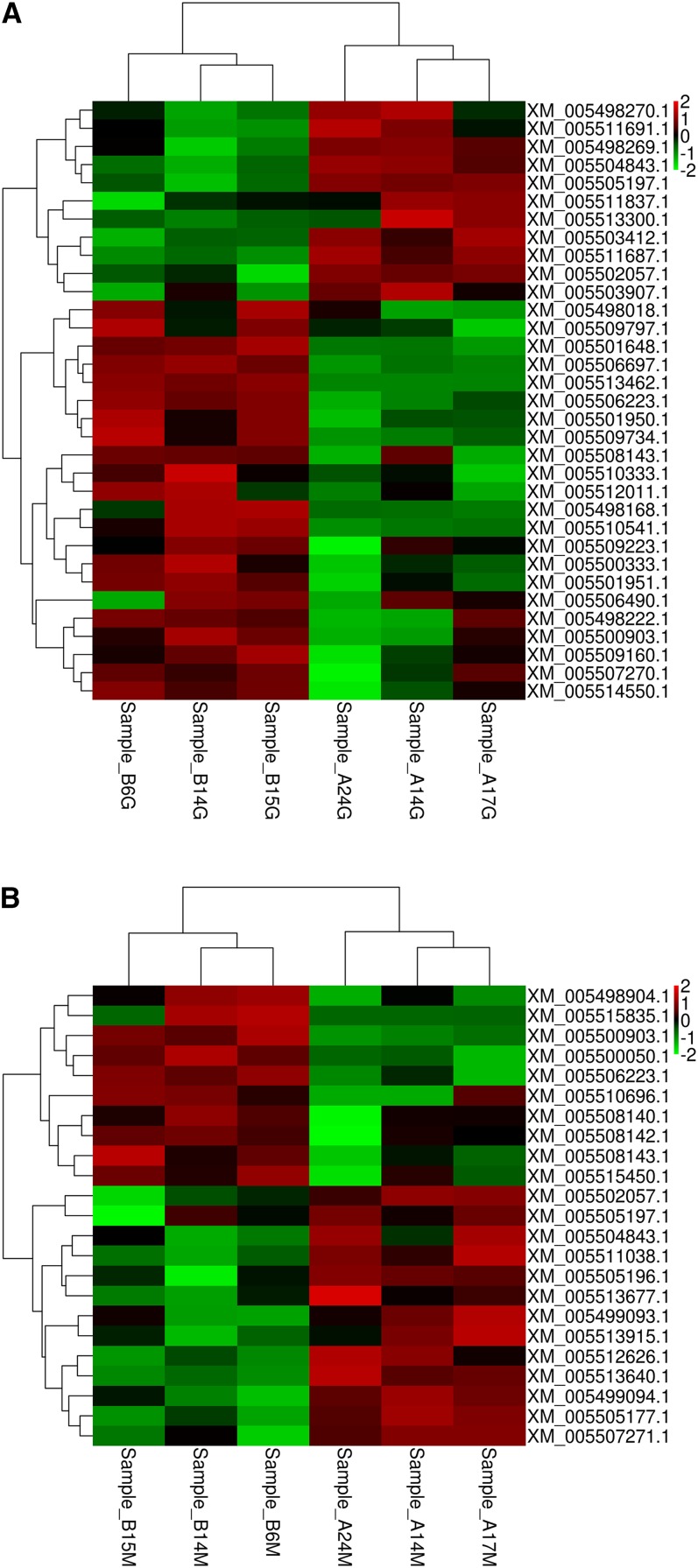
(A) Heatmap showing expression data for 33 differentially expressed transcripts in the liver tissue. Rows indicate genes with significant expression differences between the two breeds; columns represent individual samples from two pigeon breeds (Sample_A17G, Sample_A14G, Sample_A24G, and Sample_B15G, Sample_B14G, Sample_B6G were from breed A and breed B, respectively). Breed A, White Carneau; breed B, Europigeon. (B) Heatmap showing expression data for 23 differentially expressed transcripts in the muscle tissue. Rows indicate genes with significant expression differences between the two breeds; columns represent individual samples from two pigeon breeds (Sample_A17M, Sample_A14M, Sample_A24M, and Sample_B6M, Sample_B14M, Sample_B15M, were from breed A and breed B, respectively). Breed A, White Carneau; breed B, Europigeon.

Hierarchical clustering analysis was applied to compare the expression patterns of these DEGs in the liver (Figure 2A) and muscle (Figure 2B) tissues. The expression patterns of these genes can be divided into two clusters and the birds could be differentiated on the basis of the breed.

### Gene expression validation of DEGs by qRT-PCR

**Figure 3:**
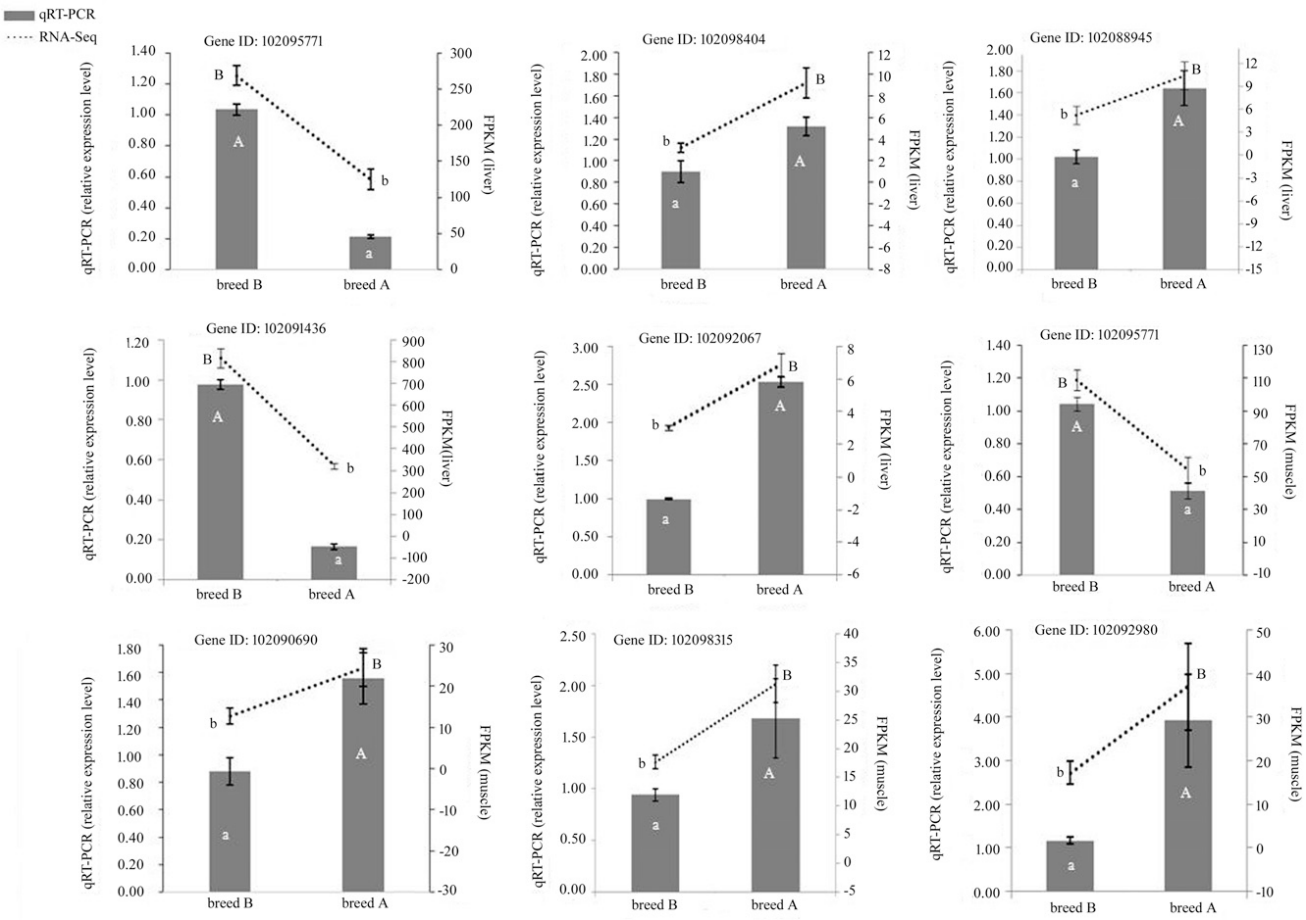
Expression of eight significant DEGs detected by RNA-Seq and validated by qRT-PCR. Results from RNA-Seq are shown by line graphs on the top and values are shown on the right Y-axis as FPKM. Results from qRT-PCR are shown by bar graphs on the bottom and values are shown on the left Y-axis as relative expression level. Breed A, White Carneau; breed B, Europigeon. “A” and “a” indicate significant difference in the relative expression of genes detected via qRT-PCR at *P* < 0.05. “B” and “b” indicate significant difference in the expression of mRNA detected from RNA-Seq at *P* < 0.05. Data are presented as mean ± SE.

In the five pathways involved in the synthesis and degradation of ketone bodies, FA biosynthesis and degradation, biosynthesis of unsaturated FAs, and arachidonic acid metabolism, we randomly selected eight DEGs to validate the expression data identified by RNA-Seq, of which, five DEGs were expressed in the liver, four DEGs were expressed in the muscle, and one was expressed in both tissues. qRT-PCR analysis confirmed the direction of changes in the expression level of DEGs detected by RNA-Seq (Figure 3), which demonstrated that data from RNA-Seq and qRT-PCR were consistent in quantitatively estimating the transcription levels of the tested transcripts.

## Discussion

In this study, squabs from two breeds had significant differences in traits regarding meat production performance and meat quality. Birds from breed A had a significantly higher proportion of breast muscle, which was favored in regard to increasing meat yields in the pigeon industry. Muscle samples from breed A also had significantly higher WHC than those from breed B. Since higher WHC is associated with reduced drip loss, better appearance of fresh meat, and better sensory properties of cooked meat (Pedersen *et al.* 2003), improvement in WHC of meat is desired both by industry and consumers. Besides these two favorable traits, the breast muscle from breed A also had significantly higher IMF content and lower WBSF value, which characterized a tender meat. A significantly negative correlation between IMF and average shear force was detected in this study, which was consistent with previous findings that demonstrated the favorable effects of increased IMF level on meat tenderness although the strength of the correlation varied between studies (Van Laack *et al.* 2001; Fortin *et al.* 2005; Teye *et al.* 2006; Jeleníková *et al.* 2008). Our results revealed that under the same rearing conditions, squabs from breed A were more efficient in increasing breast muscle yield and had better meat quality in terms of WHC, IMF content, and tenderness.

It has been widely accepted that IMF content is one of the key determinants of meat tenderness, flavor, and juiciness (Hodgson *et al.* 1991; Fernandez *et al.* 1999). Since the IMF content of meat can be influenced by both genetic and environmental factors (Dikeman *et al.* 2005), care was taken in the present study to ensure that the IMF level was assessed under conditions where other factors known to affect lipid deposition, such as slaughter age, muscle type, gender, and feeding (Dodson *et al.*, 2010; Geldenhuys *et al.* 2013; Anderson *et al.* 2015), were kept to a minimum level of variation. Therefore, breed was retained as the main source of the significant difference observed in IMF level.

It is know that IMF contributes importantly not only to various aspects of meat quality but also to the nutritional value of meat. The P/S and n-6/n-3 ratios are normally used to assess the nutritional value of fat. The recommended dietary ratios for P/S and n-6/n-3 were > 0.4 and < 4.0, respectively (Wood *et al.* 2004b). Analysis of the FA composition of IMF in the breast muscle of squabs indicated that the mean P/S ratio, 0.88 for the breast muscle, was higher than that in the breast and thigh meat of broilers, 0.54 and 0.62, respectively (Ahmed *et al.* 2015). It was also higher than the P/S ratios of other red meats, such as lamb (0.32, longissimus lumborum) (Majdoub-Mathlouthi *et al.* 2015), beef (0.44, longissimus thoracis) (Andreo *et al.* 2016), and pork (0.55−0.62, longissimus thoracis) (Alonso *et al.* 2015). As to the n-6/n-3 ratio in the breast muscle of squabs, it was also within the recommended range. It was favorably lower (2.86) in squabs compared with the n-6/n-3 ratio in beef (greater than 17.4, longissimus muscle) (Indurain *et al.* 2010), pork (greater than 9.0, longissimus thoracis) (Ayuso *et al.* 2015), broiler breast meat, and thigh meat (6.89 and 4.86, respectively) (Ahmed *et al.* 2015). Given the relationship found between incidence of coronary diseases and high ratios of n-6/n-3 FAs in meat (Santos-Silva *et al.* 2002), results from our study indicated that the breast muscle of squabs had a high ratio of P/S and a more favorable balance between n-6 and n-3 PUFA, which was beneficial and healthy for consumers.

Our results also demonstrated the better meat quality of squabs from breed A due to the higher IMF content (tender meat) and favorable FA composition (higher MUFA and lower SFA content), which met the recommendation of lowering the dietary intake of SFA and increasing that of unsaturated FAs to decrease the risk of cardiovascular diseases (Simopoulos 2002; Gidding *et al.* 2005). This breed-specific difference in meat quality might be used for selection programs in the pigeon industry to produce a product that has enough IMF to ensure a pleasant eating experience and at the same time alleviate the health concerns associated with high-SFA meat. Further research needs to be carried out to determine whether the inclusion of breed A in the parental line will result in an increase in IMF and improvement in the FA profile in the offspring to make the squab meat more attractive for health reasons.

In order to identify genes closely associated with the genetic variation in IMF deposition and FA composition in squabs, transcriptomes of the breast muscle and liver tissue were compared between squabs from two pigeon breeds which differed significantly in these traits. Of all the significant DEGs involved in lipid metabolism, several functional genes have been previously reported to be prosperous candidate genes related to IMF trait or FA composition in livestock, including *ADH* (alcohol dehydrogenase 1) (Ward *et al.* 2012), *ANGPTL4* (angiopoietin-like 4) (Ren *et al.* 2014), *FADS1* (fatty acid desaturase 1) (Ibeagha-Awemu *et al.* 2014), *ACSL4* (acyl-CoA synthetase long-chain family member 4) (Ruść *et al.*, 2011; Chen *et al.* 2014), and *LPL* (lipoprotein lipase) (Wang *et al.* 2013; Zhang *et al.* 2015). However, the majority of significant DEGs involved in lipid metabolism have not been documented to be associated with the differences in the capacity of lipid accumulation. Interestingly, the widely reported *FABP* genes, including the muscle-and-heart type (*FABP3*), adipocyte type (*FABP4*), and liver type (*FABP1*), which have been revealed to be significantly related to the variations of IMF level in pig (Zhao *et al.* 2009; Lee *et al.* 2010; Han *et al.* 2012), chicken (Ye *et al.* 2010; Wang *et al.* 2016), duck (He *et al.* 2012), and milk FA composition in dairy cattle (Nafikov *et al.* 2013), were not identified as significant DEGs in this study although the mRNA expression levels of *FABP3*, *FABP4*, and *FABP1* in the liver tissues of breed A were higher than those of breed B (with an up-regulated fold change of 1.10, 1.97, and 4.73, respectively). These results led us to suggest that either FABP might not regulate the FA metabolism at the transcriptomic level or there might be digestive, nutritional, or metabolic particularities between squabs and other meat-producing animals. Further studies need to screen potential SNPs in these DEGs in different pigeon populations to assess possible associations between genetic variability and IMF content, as well as the FA profiles in meat before markers identified might be used in improvement programs aiming to produce a more competitive product with tender squab meat and favorable FA composition.

We also focused on significant DEGs identified in the PPAR signaling pathway because there is increasing evidence suggesting the involvement of this pathway in lipid metabolism (Doran *et al.* 2014; Hausman *et al.* 2014; Zheng *et al.* 2015). Association studies between polymorphisms in genes in the PPAR signaling pathway and porcine meat quality traits have been carried out (He *et al.* 2013) and the positive correlation between IMF deposition and PPAR signaling genes has been reported in chicken (Cui *et al.* 2012). In this study, we found that seven of the ten unique genes identified in the PPAR signaling pathway were significantly up-regulated in the high-IMF breed A, including three genes involved in acyl-CoA synthesis, *ACSL4*, *ACSL5* (acyl-CoA synthetase long-chain family member 5), and *ACSBG2* (acyl-CoA synthetase bubblegum family member 2), indicative of an enhanced lipogenesis. Our results were in agreement with previous studies that documented these genes as potential candidate genes associated with the difference in lipid metabolism (Pan *et al.* 2010; Corominas *et al.* 2012; Claire D’Andre *et al.* 2013). While *ACSBG1* (acyl-CoA synthetase bubblegum family member 1), the fourth gene encoding an acyl-CoA synthetase, was up-regulated in low-IMF breed B, its significant up-regulation in skeletal muscle was observed in mice administered metformin, which suppressed lipid accumulation by promoting FA oxidation (Wang *et al.* 2014). We suggested that these genes may form the foundation for further investigation to identify causative mutations involved in fat deposition and FA composition in the muscle of squabs. Also, future studies on the translational level of proteins encoded by these DEGs as well as information about the expression changes due to different variants would be needed to elucidate their effects on lipid metabolism in squabs.

### Conclusions

In this study, we demonstrated that squabs from two meat pigeon breeds, White Carneau and Europigeon, had significant differences in meat traits with regard to WHC, tenderness, IMF content, and FA composition. Datasets from transcriptome profiling of the breast muscle and liver tissue were generated and functional genes differentially expressed between the high- and low-IMF squabs were identified, among which, genes involved in lipid metabolism and the PPAR signaling pathway might be potential candidate genes associated with the variation of IMF content and FA composition in the muscle of squabs. These genes will help to identify DNA markers to predict the ability of squabs to deposit IMF and use them in selection programs in order to provide squab with enhanced meat quality and ensure the competitiveness of the pigeon industry.

## Supplementary Material

Supplemental Material
