# Supplementary material for: Transcriptomic Analysis Identifies Candidate Genes Related to Intramuscular Fat Deposition and Fatty Acid Composition in the Breast Muscle of Squabs (Columba)
Source: G3 (Bethesda). 2016 May 9;6(7):2081–90. doi: 10.1534/g3.116.029793 (PMC4938661; doi:10.1534/g3.116.029793)
Supplement: Supplemental Material [file supp_6_7_2081__index.html]

Transcriptomic Analysis Identifies Candidate Genes Related to Intramuscular Fat Deposition and Fatty Acid Composition in the Breast Muscle of Squabs (Columba) — Supplemental Material 

# Transcriptomic Analysis Identifies Candidate Genes Related to Intramuscular Fat Deposition and Fatty Acid Composition in the Breast Muscle of Squabs (*Columba*)

## Supplemental Material for Ye *et al.*, 2016

**Files in this Data Supplement:**

- Table S1 - Nucleotide sequences of primers used for qRT-PCR. (.pdf, 159 KB)
- File S1 - The significantly enriched GO terms in the liver tissue. (.txt, 504 KB)
- File S2 - The significantly enriched GO terms in the muscle tissue. (.txt, 435 KB)
- File S3 - The detected significantly expressed genes involved in the lipid metabolism and the PPAR pathway. (.xls, 27 KB)
