# Supplementary material for: Transcriptomic Analysis Identifies Candidate Genes Related to Intramuscular Fat Deposition and Fatty Acid Composition in the Breast Muscle of Squabs (Columba)
Source: G3 (Bethesda). 2016 May 9;6(7):2081–90. doi: 10.1534/g3.116.029793 (PMC4938661; doi:10.1534/g3.116.029793)
Supplement: Supplemental Material [file supp_g3.116.029793_TableS1.pdf]

Table S1. Nucleotide sequences of primers used for qRT-PCR.

ID	Gene	Expression	Sequence (5’ to 3’)	Size of amplicon (bp)
XM_005506223.1	hydroxysteroid (17-beta) dehydrogenase 4 (*HSD17B4*)	Liver and muscle	up: gctcccctagttgtttggct<br>down: acttccaggtttgctggcat	207
XM_005503412.1	leukotriene C4 synthase-like (LOC102098404)	Liver	up: ctgcagccttgggtctactc<br>down: aagctggaggatgttcagcc	191
XM_005511837.1	acyl-CoA synthetase long-chain family member 4 (*ACSL4*)	Liver	up: ctacggctgcaggattggat<br>down: aggtgcatcgtatcccctct	250
XM_005506697.1	aldehyde dehydrogenase 7 family member A1 (*ALDH7A1*)	Liver	up: ccattgtgactggccttcct<br>down: gtcagatccctttggcccaa	197
XM_005511687.1	3-hydroxybutyrate dehydrogenase type 1 (*BDH1*)	Liver	up: ctgcctgcggtatgaaatgc<br>down: ccatcttgctgacctgctca	194
XM_005513915.1	acyl-CoA synthetase bubblegum family member 2 (*ACSBG2*)	Muscle	up: tgaacgggtgctctgaagtc<br>down: acgggaatgttcaggctcag	166
XM_005511038.1	fatty acid desaturase 1-like (*FADS1*)	Muscle	up: gcttggctcgtcatctggta<br>down: cagttgggtttggcatggtg	239
XM_005513677.1	lipocalin-like (LOC102092980)	Muscle	up: cagacatctccaccactgca<br>down: tgaacaccatggcgtactcc	198
XM_005506273.1	glyceraldehyde-3-phosphate dehydrogenase (*GAPDH*)	Muscle	up: tgaaagtcggagtcaacggat<br>down: acgctcctggaagatggtgat	230
